# A Decade of Change: National Trends in Management and Outcomes of Achalasia Hospitalizations in the United States

**DOI:** 10.1111/nmo.70202

**Published:** 2025-11-03

**Authors:** Ritik M. Goyal, Anand Shah, Rohan Karkra, Sameer Rao, Amanda Rupert

**Affiliations:** ^1^ Department of Internal Medicine Rutgers New Jersey Medical School Newark New Jersey USA; ^2^ Division of Gastroenterology and Advanced Endoscopy, Department of Internal Medicine Rutgers New Jersey Medical School Newark New Jersey USA

**Keywords:** achalasia, laparoscopic Heller's myotomy, POEM, trends

## Abstract

**Background:**

Achalasia is a rare esophageal motility disorder managed through various interventions, including laparoscopic Heller myotomy (LHM), pneumatic dilation (PD), and, more recently, per‐oral endoscopic myotomy (POEM). Over the past decade, there has been a shift toward POEM, though nationwide hospitalization trends and outcomes remain understudied.

**Methods:**

We conducted a retrospective trend analysis using the National Inpatient Sample (NIS) database from 2011 to 2022. Adult patients hospitalized with a primary diagnosis of achalasia were identified using ICD‐9/10‐CM codes. Trends in management strategies (LHM, POEM, PD, and esophagectomy), hospitalization outcomes, and complications were analyzed. Multivariable regression adjusted for patient and hospital characteristics was also performed to compare outcomes between 2011 and 2022.

**Results:**

A total of 63,420 weighted achalasia‐related admissions were identified. The use of LHM declined significantly (AAPC −4.66%, *p* < 0.001), while POEM use rose from 5.8% in 2016 to 10.3% in 2022 (AAPC 7.76%, *p* = 0.049). Hospitalization costs increased annually by 3.18% (*p* < 0.001), while the length of stay remained stable. Mortality rates rose over time; however, there was no difference after adjusting for patient‐related factors. Adjusted analyses revealed increased odds of POEM in 2022 (aOR 1.87), decreased odds of LHM and esophagectomy, and higher odds of respiratory complications.

**Conclusions:**

From 2011 to 2022, there has been a clear shift toward the use of POEM for the inpatient management of achalasia. However, worsening patient comorbidity profiles have driven increased complications and mortality and may contribute to increasing costs. Early intervention and broader POEM adoption may optimize future outcomes.

## Graphical abstract

From 2011 to 2022, inpatient management of achalasia declined while hospitalization costs rose. POEM utilization increased, accompanied by declines in LHM and esophagectomy rates. As patients presented with higher comorbidity burdens, both mortality and respiratory complications showed an upward trend.

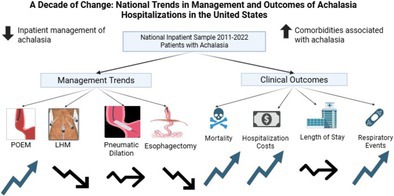

1

**Summary**

- Evolving management trends: from 2011 to 2022, inpatient management of achalasia in the United States has shifted toward the use of minimally invasive endoscopic procedures such as per‐oral endoscopic myotomy (poem), with a concurrent decline in traditional surgical approaches like laparoscopic Heller myotomy.
- Decline in inpatient interventions: overall inpatient management for achalasia have decreased over the past decade, reflecting a transition toward outpatient‐based management as POEM become more widely adopted.
- Increasing comorbidity burden: hospitalized patients with achalasia now present with more complex comorbidities than in prior years, which may contribute to the observed rise in complications, mortality, and healthcare costs despite stable lengths of stay.

## 2 Introduction

Achalasia is a rare motility disorder of the esophagus characterized by impaired relaxation of the lower esophageal sphincter (LES) and dysfunctional peristalsis. Globally, the prevalence is estimated to be 10.82 cases per 100,000 persons, with approximately 20,000–40,000 cases within the United States (US) [1]. This condition affects men and women equally, with a peak incidence between 30 and 60 years of age. It leads to progressively bothersome symptoms such as heartburn, chest pain, progressive dysphagia, regurgitation, weight loss, and nutritional deficiencies [2].

Achalasia is a chronic and progressive condition without a definitive cure. Management aims at symptomatic relief by reducing LES pressure and relieving the physiological obstruction at the gastroesophageal junction. Current treatment options include pneumatic dilation (PD), laparoscopic Heller's myotomy (LHM), and per‐oral endoscopic myotomy (POEM) [3]. Botulinum toxin injection to the LES may be effective but is of limited duration, and pharmacologic agents like calcium channel blockers (CCB), anticholinergics, and nitrates have been tried with questionable benefits [3]. Esophagectomy is reserved as a last‐resort intervention for those with advanced disease and/or who have failed other therapies, although this is associated with significant morbidity [4].

POEM is a minimally invasive endoscopic procedure that has gained popularity since its first human case in 2008. POEM was introduced in the US in 2010 by Stavropoulos [5]. This procedure involves creating a submucosal tunnel and performing a myotomy of the LES using an endoscope. This technique allows for a longer myotomy, which can be beneficial for patients with type III achalasia [6]. The American Society for Gastrointestinal Endoscopy (ASGE) suggests POEM as the preferred treatment for type III achalasia due to its efficacy in relieving symptoms [7]. Considering the advances in the management of achalasia and the emergence of POEM, we aim to study the evolving trends regarding the preferred interventions for the management of achalasia as well as related hospitalization outcomes and complications.

## 3 Methods

We performed a retrospective descriptive trend analysis using the Healthcare Cost Utilization Project's (HCUP's) National Inpatient Sample (NIS) from the years 2011 to 2022. NIS provides a 20% stratified sample of all the discharges from participating US community hospitals spread over 48 states and includes 97% of the US population. The NIS contains deidentified clinical and non‐clinical data, including patient demographics, total charges, length of stay, diagnoses, and procedures performed during hospital stays. Diagnoses and procedures are coded using the International Classification of Diseases, Ninth Revision, Clinical Modification (ICD‐9‐CM) and Procedure Coding System (ICD‐9‐PCS) before October 2015 and the ICD‐10‐CM and ICD‐10‐PCS codes thereafter (Supporting Information). As the NIS data are fully deidentified, this study was exempt from the institutional review board (IRB) approval in accordance with federal regulations. All analyses were conducted in compliance with the HCUP data use agreement [8].

We identified patients admitted with the primary diagnosis of achalasia using the ICD‐9‐CM code 530.0 and ICD‐10‐CM code K22.0. Hospitalization was defined as an inpatient admission recorded in the HCUP‐NIS dataset. NIS includes only discharges from hospital admissions, irrespective of length of stay; same‐day admissions and discharges (zero‐night stays) are rare but may be included. According to the HCUP protocol, discharge weights were applied to the cohort to generate nationally representative estimates. All findings in this study are presented as nationally weighted estimates [9]. Patient‐specific variables extracted included age, sex, race, insurance type, and discharge disposition. Comorbidity severity was assessed using the Charlson comorbidity index (CCI), which calculates estimated 10‐year survival based on the number of comorbidities present [10]. Further, ICD‐9 and ICD‐10 procedure codes were utilized to identify LHM, PD, POEM, and esophagectomy. The percentages of patients undergoing LHM, PD, and esophagectomy were calculated for the entire study period (2011–2022), while the proportion of patients undergoing POEM was assessed from 2016 to 2022, as ICD codes were unavailable for POEM before 2016. Outcomes studied included mortality, length of stay, total hospital charges (adjusted for consumer price index for medical care as per 2011 rates), and complications such as aspiration pneumonia and respiratory failure.

Time trends were reported as annual percentage change (APC), which reflects the change in rates between two subsequent years, and average APC (AAPC), which reflects the average change between the incidence rates over the entire study period (2011–2022). The trends were generated using Joinpoint Regression Software (v.5.0.2, NCI) via the weighted Bayesian Information Criteria (BIC) methodology [11, 12]. Data analysis was done using the IBM SPSS software. A *p*‐value < 0.05 was considered significant.

We conducted a multivariate regression analysis to compare the management strategies and outcomes for achalasia between 2011 and 2022 (2016–2022 for POEM). The outcomes were adjusted for patient‐level factors including age, sex, race, insurance status, and comorbidities based on the CCI, as well as hospital‐level characteristics including teaching status, location and setting. Logistic regression was used for categorical variables, while linear regression was used to analyze the continuous variables (LOS and hospital charges). Given multiple outcomes were tested, *p*‐values were adjusted using the Benjamini–Hochberg false discovery rate (BH‐FDR) procedure across the 11 primary outcomes reported in Table 4 (*q* = 0.05). Results were interpreted after BH‐FDR correction.

## 4 Results

We identified 12,740 patients admitted to the hospital primarily for achalasia from 2011 to 2022, which amounts to 63,420 hospital admissions when weighted and accounts for 17.69 per 100,000 total admissions. The mean age over the time period of study was 60.4 years, and 46.72% of the study sample was male. The patients admitted with a CCI score of ≥ 3 increased from 13.6% to 28.1% (*p* < 0.001) between 2011–2012 and 2021–2022. For the entire study period, most individuals were insured with Medicare; however, the percentage of patients with Medicare increased from 44% to 53.9% between 2011–2012 and 2021–2022. Detailed two‐year demographic characteristics of the patients are provided in Table 1.

**TABLE 1:** Demographic, comorbidity profile, and outcomes of patients admitted with achalasia.

	Total	2011–2012	2013–2014	2015–2016	2017–2018	2019–2020	2021–2022	*p*
Number of achalasia admissions (*N*)	12,740	2192	2110	2284	2210	2025	1919	
Weighted *N*	63,420	10,680	10,550	11,420	11,050	10,125	9595	
Prevalence per 100,000	17.687	16.84	17.66	18.92	18.21	17.45	17.04	
Age (mean)	60.4	59.12 ± 18.68	58.75 ± 18.86	59.48 ± 18.29	60.61 ± 17.98	61.93 ± 17.90	62.73 ± 17.55	0.009
Female (%)	53.25	52.93	54.78	53.52	53.84	52.74	51.43	0.388
Race (%)								0.049
Whites	67.49	69.97	68.74	67.08	65.88	66.09	67.19	
Blacks	16.48	16.38	15.78	16.52	18.28	16.95	14.74	
Hispanics	10.45	8.85	10.06	10.63	10.47	11.13	11.60	
Asian	2.08	1.76	1.51	1.99	2.49	2.17	2.55	
Native American	0.35	0.39	0.40	0.37	0.37	0.20	0.31	
Others	3.16	2.64	3.49	3.38	2.49	3.44	3.56	
CCI score, %								< 0.001
0	57.00	60.99	59.85	59.10	56.10	52.98	52.11	
1	10.94	14.32	15.54	10.24	8.00	9.18	9.12	
2	10.94	11.04	10.14	10.20	11.76	10.96	11.62	
≥ 3	21.98	13.64	14.45	20.44	24.11	26.86	28.13	
Insurance type (%)								< 0.001
Medicare	48.88	44.0	45.4	46.3	50.7	53.9	53.9	
Medicaid	10.84	9.6	9.5	11.6	11.2	11.6	11.5	
Private insurance	33.84	38.8	37.7	36.0	32.4	29.4	27.7	
Self‐pay	3.11	3.4	3.8	3.1	3.2	2.5	2.6	
No charge	0.39	0.7	0.6	0.4	0.2	0.2	0.2	
Other	2.95	3.4	3	2.6	2.3	2.3	4.1	
Discharge disposition (%)								< 0.001
Home	77.84	80.78	79.71	79.80	76.33	75.20	74.57	
Short‐term hospital	2.02	1.5	1.65	2.01	2.44	2.12	2.39	
Facility	7.71	7.39	6.91	7.79	8.64	8.04	7.39	
Home health care	11.12	9.44	10.42	9.46	11.26	13.03	13.60	
AMA	0.7	0.45	0.61	0.52	0.63	0.83	1.19	
Mortality	0.6%	0.41%	0.61%	0.39%	0.63%	0.74%	0.83%	
Hospitalization charges (in USD)		44380.51	49552.96	57,509.60	62772.34	66251.38	74446.48	0.0029
Length of stay (in days)	4.24	4.13	4.14	4.30	4.18	4.27	4.39	0.129
Management (%)								
Heller's myotomy	38.42	45.30	44.55	42.08	36.47	31.26	29.29	
POEM (2016–2022)	8.27				8.10	8.40	9.80	
Pneumatic dilatation	5.71	6.39	8.91	5.21	3.48	4.94	5.37	
Esophagectomy	0.94	1.46	1.47	1.18	0.50	0.44	0.52	

### 4.1 Management Trends

**FIGURE 1:**
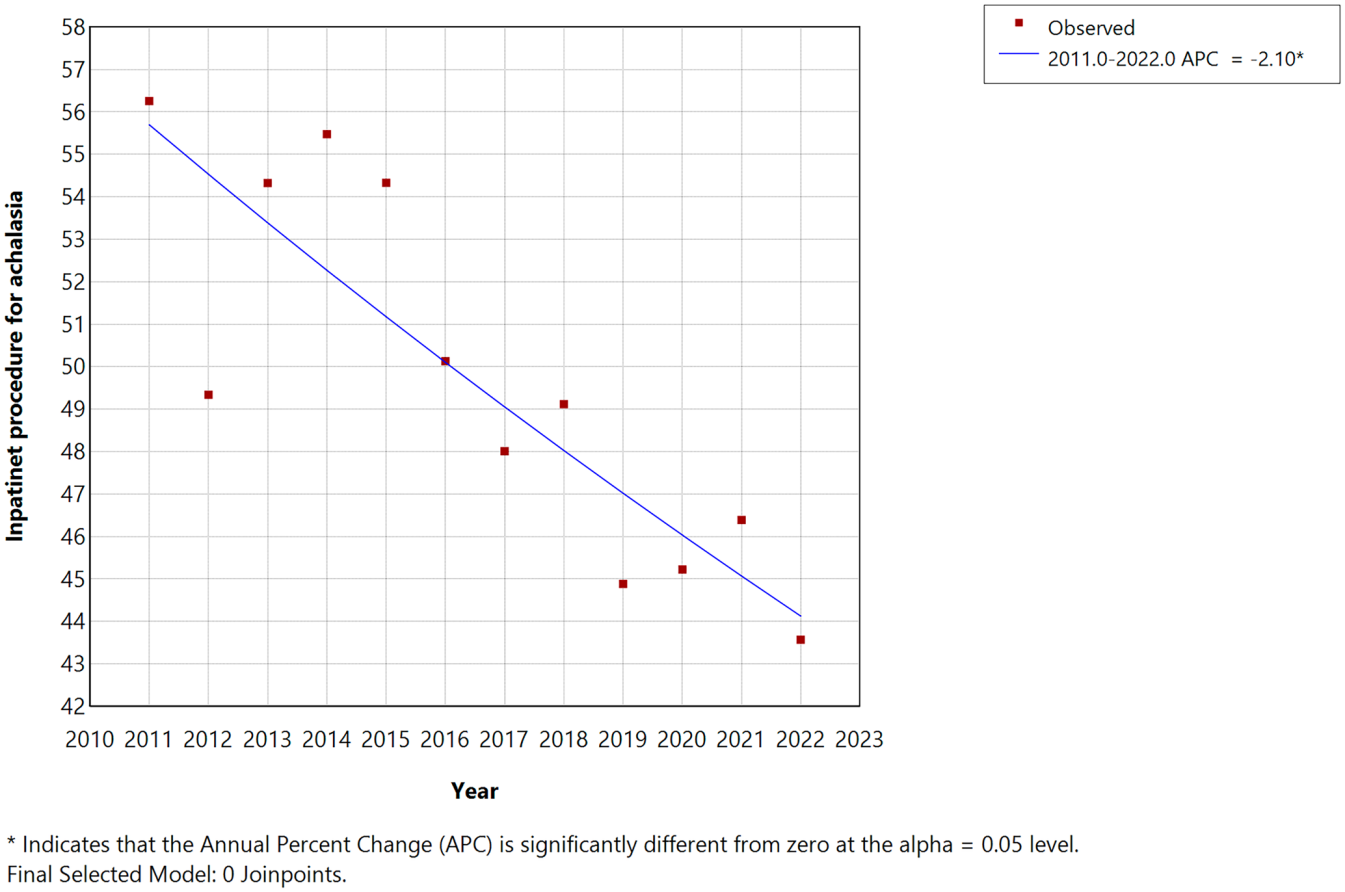
Trends in overall inpatient management of achalasia in the United States (2011–2022). This figure demonstrates a steady decline in the overall rates of inpatient management for achalasia over the last decade.

**FIGURE 2:**
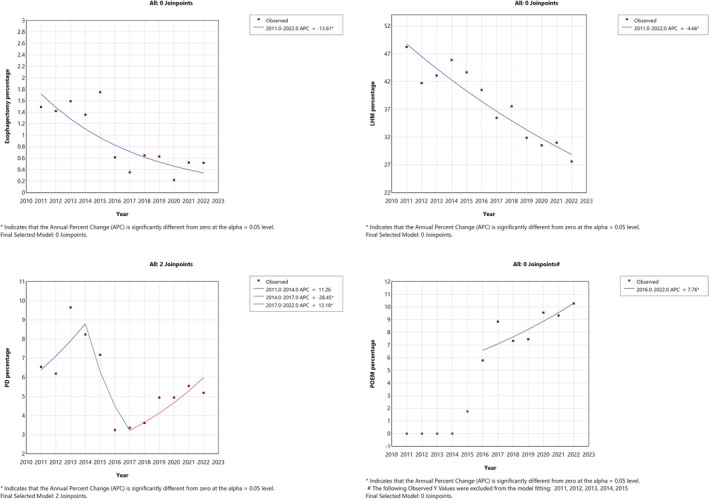
Trends in utilization of achalasia treatments in the United States, 2011–2022. This figure presents annual trends in the use of esophagectomy, laparoscopic Heller myotomy (LHM), pneumatic dilation (PD), and per‐oral endoscopic myotomy (POEM) based on percentage utilization per year from 2011 to 2022. Each panel shows observed data points and the modeled trend line based on Joinpoint regression analysis.

Over the years, there has been a decline in the inpatient management of achalasia, with 56.2% of patients managed in the hospital setting in 2011 compared to only 43.6% in 2022, corresponding to an AAPC of −2.1% (95% CI: −3.0% to −1.2%, *p* < 0.001) (Figure 1). There was a decrease in using LHM as the management modality for patients admitted with achalasia. In 2011–2012, the percentage of patients undergoing LHM was 45.3%, which dropped to 29.3% in 2021–2022 with an AAPC of −4.7% (95% CI: −6% to −3.2%, *p* < 0.001). Similarly, esophagectomy also declined, with 1.5% of achalasia patients undergoing esophagectomy in 2011–2012 to only 0.5% in 2021–2022, with an AAPC of −13.6% (95% CI: −20.1% to −6.7%, *p* < 0.001). Meanwhile, the proportion of patients undergoing POEM increased between 2016 and 2022 from 5.8% to 10.3%, with an AAPC of 7.8% (95% CI: 0.02%–16.4%, *p* = 0.049) (Table S2). However, the proportion of achalasia patients undergoing pneumatic dilation remained stable from 2011 to 2014 (*p* = 0.116), declined from 2014 to 2017 (APC: −48.4%, 95% CI: −37.4% to −13.4%, *p* = 0.005), and increased from 2017 to 2022 (APC = 13.2%, 95% CI: 3%–44.4%, *p* = 0.014) (Table 2; Figure 2).

**TABLE 2:** Management trends of patients admitted with achalasia from 2011 to 2022.

Procedure	Year	APC	*p*	AAPC	*p*
Any inpatient procedure	2011–2022	−2.1 (−3 to −1.2)	< 0.001	−2.1 (−3 to −1.2)	< 0.001
LHM	2011–2022	−4.66 (−6 to −3.32)	< 0.001	−4.66 (−6 to −3.32)	< 0.001
POEM	2016–2022	7.76 (0.02–16.41)	0.049	7.76 (0.02 to 16.41)	0.049
Pneumatic dilation	2011–2014	11.26 (−3.31 to 54.04)	0.116	−0.59 (−4.12 to 4.2)	0.869
	2014–2017	−48.45 (−37.43 to −13.42)	0.005		
	2017–2022	13.18 (3.02–44.39)	0.014		
Esophagectomy	2011–2022	−13.61 (−20.13 to −6.75)	< 0.001	−13.61 (−20.13 to −6.75)	< 0.001

### 4.2 Hospitalization Outcomes and Complications

**FIGURE 3:**
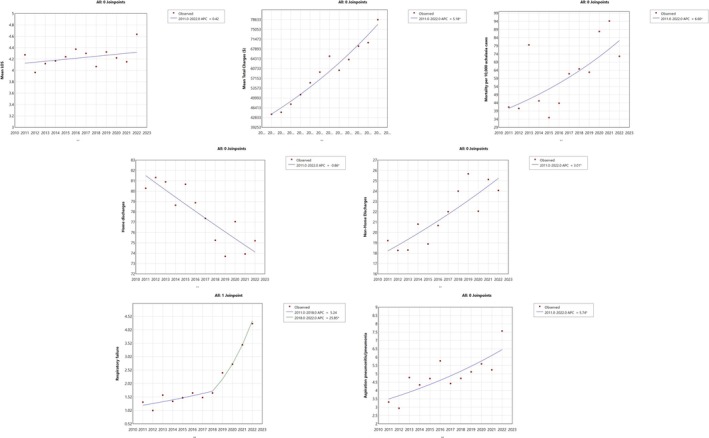
Trends in clinical and healthcare outcomes among achalasia hospitalizations in the United States, 2011–2022. This figure displays Joinpoint regression analyses of temporal trends in key hospitalization‐related outcomes among patients with achalasia from 2011 to 2022.

Over the years 2011–2022, the total hospitalization cost increased with an APC and AAPC of 3.18% (95% CI: 2.1%–4.2%, *p* < 0.001). The mean length of stay in the hospital was 4.23 days for the entire cohort and remained stable over the years (*p* = 0.332). A significant increase in mortality was noted from 0.4% to 0.8% between 2011 and 2022, with an APC and AAPC of 6.6% (95% CI: 2.1%–10.9%, *p* = 0.003) for mortality per 10,000 achalasia cases. The number of patients with achalasia discharged to home decreased (APC: −0.9%, 95% CI: −1.3% to −0.4%, *p* < 0.001), while there was an increase in the number of patients being discharged to facility or with home care (APC: 3%, 95% CI: 1.2%–4.8%, *p* = 0.002). At the same time, there was an increasing trend in the respiratory complications associated with achalasia, such as aspiration pneumonitis or pneumonia, from 2011 to 2022 (APC: 5.7%, 95% CI: 1.7%–9.8%, *p* = 0.005) and respiratory failure from 2018 to 2022 (APC: 25.8%, 95% CI: 15.1%–50.9%, *p* < 0.001) (Table 3; Figure 3). Although we examined additional complications such as bleeding, perforation, and sepsis, the number of events was too low to report (< 10), in accordance with NIS reporting guidelines, which limits our ability to comment on these outcomes.

**TABLE 3:** Hospitalization outcomes and complications trends in patients admitted with achalasia from 2011 to 2022.

Outcome	Year	APC	*p*	AAPC	*p*
Length of stay	2011–2022	0.42 (−0.46 to 1.24)	0.332	0.42 (−0.46 to 1.24)	0.332
Total hospitalization charges^a^	2011–2022	3.18 (2.15–4.21)	< 0.001	3.18 (2.15–4.21)	< 0.001
Mortality per 10,000 cases	2011–2022	6.6 (2.1–10.94)	0.003	6.6 (2.1–10.94)	0.003
Aspiration pneumonia or pneumonia	2011–2022	5.74 (1.71–9.77)	0.005	5.74 (1.71–9.77)	0.005
Respiratory failure	2011–2018	5.24 (−4.78 to 9.28)	0.184	12.31 (9.19–14.91)	< 0.001
	2018–2022	25.85 (15.1–50.91)	< 0.001		
Non‐home discharge	2011–2022	3.01 (1.21–4.83)	0.002	3.01 (1.21–4.83)	0.002
Home discharge	2011–2022	−0.86 (−1.3 to −0.42)	< 0.001	−0.86 (−1.3 to −0.42)	< 0.001

^a^ Adjusted for consumer price index for medical care as per 2011 rates.

### 4.3 Multivariate Regression Analysis

Compared to the reference year 2016, patients in 2022 had significantly higher odds of undergoing POEM with an adjusted odds ratio (aOR) of 1.9 (95% CI: 1.3–2.6, *p* < 0.001). Additionally, compared to the reference year 2011, the odds of undergoing LHM were significantly lower (aOR: 0.4, 95% CI: 0.3–0.5, *p* < 0.001). The use of pneumatic dilation did not differ significantly between the years (aOR: 0.8, 95% CI: 0.5–1.2, *p* = 0.21), while the odds of esophagectomy were significantly lower in 2022 (aOR: 0.3, 95% CI: 0.1–0.8, *p* = 0.017). Among clinical outcomes, respiratory failure (aOR: 2.84, 95% CI: 1.54–5.24, *p* = 0.001) and aspiration pneumonitis (aOR: 2.09, 95% CI: 1.38–3.17, *p* < 0.001) were more likely to occur in the year 2022. There were no significant differences in the odds of sepsis (*p* = 0.992) or home discharge (*p* = 0.303) between the two time periods. The adjusted total hospital charges were significantly higher by 69.4% (95% CI: +58.5 to +81.2%, *p* < 0.001); however, the length of stay (*p* = 0.748) and mortality (*p* = 0.958) had no significant change between the years 2011 and 2022 (Table 4). After applying for the BH‐FDR correction, there was no change in the level of statistical significance across the 11 tested outcomes.

**TABLE 4:** Multivariate analysis comparing outcomes in the year 2022 (reference year—2011), adjusted for age, sex, insurance status, race, CCI score, and hospital‐level factors including teaching status, location and setting.

Outcome	Adjusted OR/*β*	95% CI	*p* ^b^
POEM^a^	1.87	1.33–2.62	< 0.001
LHM	0.42	0.34–0.51	< 0.001
Pneumatic dilation	0.78	0.53–1.15	0.21
Esophagectomy	0.26	0.09–0.79	0.017
Respiratory failure	2.84	1.54–5.24	0.001
Sepsis	0.99	0.4–2.5	0.992
Aspiration pneumonitis	2.09	1.38–3.17	< 0.001
Home discharge	0.89	0.7–1.12	0.303
Mortality	1.03	0.32–3.35	0.958
Length of stay	0.09 days	−0.45 to +0.63	0.748
Hospital charges	69.4%	+58.5 to +81.2%	< 0.001

^a^ Year 2016 as reference.

^b^ BH‐FDR correction applied across all 11 primary outcomes; POEM, LHM, respiratory failure, aspiration pneumonitis, esophagectomy, and hospital charges remained significant.

## 5 Discussion

This study evaluated the trends in achalasia‐related admissions, management strategies, and clinical outcomes from 2011 to 2022. Over this period, the proportion of patients undergoing inpatient achalasia management has decreased. We observed significant shifts in management practices, with a notable rise in the use of minimally invasive techniques such as POEM and a decline in traditional surgical approaches like LHM. Additionally, we documented a decrease in the utilization of esophagectomy. However, the comorbidity profile of hospitalized achalasia patients has progressively worsened over the past decade. This deterioration is associated with poorer hospitalization outcomes, including increased mortality rates, higher hospitalization costs, and more frequent need for medical care at the time of discharge either in a rehabilitation facility or in the home. These findings underscore the need for continued efforts to optimize the management of achalasia in increasingly comorbid populations.

Over the past decade, achalasia management has increasingly shifted to outpatient settings, with POEM and PD now commonly performed as same‐day procedures [3]. In our analysis, we observed a decreasing proportion of hospitalized patients with achalasia undergoing procedural interventions. Specifically, there was a declining trend in the use of LHM and esophagectomy. In contrast, the proportion of patients undergoing POEM increased markedly from 2016 to 2022. However, this increase did not fully offset the decline in LHM, suggesting that the majority of POEM procedures are being performed in outpatient settings and are therefore not captured in our inpatient dataset. This shift is supported by an increasing number of gastroenterologists who recognize POEM as a primary treatment for achalasia due to its minimally invasive nature and high success rates despite a higher incidence of gastroesophageal reflux. This is further corroborated by a randomized trial published by Werner et al., which demonstrated the noninferiority of POEM compared to LHM in controlling achalasia symptoms [13]. A meta‐analysis by Akintoye et al. also found that POEM has a 98% success rate [14]. A crucial barrier to the wide‐scale adoption of POEM has been the higher learning curve and technical difficulty. A study by Patel et al. found that 40 POEMs are needed to achieve efficiency and 60 to attain mastery [15]. However, increasing education and training in this technique likely explains the rising use of POEM in managing achalasia. Moreover, a continued rise in the use of POEM is expected, as both the American College of Gastroenterology (ACG) and the American Society for Gastrointestinal Endoscopy (ASGE) recommend POEM as first‐line treatment for all subtypes of achalasia, whereas LHM and PD are recommended primarily for subtypes I and II [3, 7].

A study by Trieu et al. compared achalasia‐related hospitalizations in 2017 versus 2013 using the NIS database. They reported a decline in the use of LHM and PD, along with higher odds of pneumonia and sepsis in achalasia patients in 2017 compared to 2013 [16]. However, drawing meaningful conclusions from only two time points is limited. In contrast, our study offers a comprehensive 12‐year trend analysis of achalasia‐related hospitalizations and associated outcomes.

During the study period, the total hospitalization cost for achalasia patients increased by an average of 3% annually. This increase in cost is consistent with the findings of Gaber et al., who reported high healthcare utilization rates and a significant economic burden associated with achalasia, with estimates of costs exceeding $408 million in 2018 [17]. At the same time, the mean length of hospital stay was stable at around 4 days for the entire study period. This stability in hospital stay duration contradicts the findings of Haisley et al., who noted a decrease in hospital length of stay for achalasia patients undergoing Heller myotomy over a 20‐year period [18].

More importantly, a significant increase in the mortality rate was noted from 2011 to 2022 (AAPC: 6.6%, *p* = 0.003). This increase is concerning and related to the increasing trend of comorbidity profiles and higher incidence of associated morbidities such as aspiration pneumonia and respiratory failure, as also reported by Harvey et al. in a study from England [19]. Notably, after adjusting for comorbidities and demographic factors, the odds of mortality in 2022 were not significantly different from those in 2011. This finding suggests that the rise in mortality among patients with achalasia is not due to the disease or management itself, but rather to the worsening comorbidity profile of the affected population. While the number of achalasia patients discharged to home decreased and discharges to a facility or with home healthcare increased over time, this trend was also not statistically significant in the adjusted multivariable regression analysis. Moreover, as the proportion of patients undergoing procedures during hospitalization has declined and achalasia is increasingly managed in outpatient settings, this introduces a selection bias when evaluating trends in complications and outcomes, since hospitalized patients are more likely to represent a sicker population with a greater burden of comorbidities.

This study is the first to examine trends in hospitalization for achalasia within the US population, offering valuable insights over a 12‐year span. By analyzing nationwide data, we provide a comprehensive evaluation of the adoption of newer management modalities, such as the increasing use of POEM, while also documenting the decline in more traditional surgical approaches. Additionally, our study offers a detailed assessment of the complications associated with achalasia, along with other key hospitalization outcomes making it a significant contribution to the current understanding of achalasia management trends.

Despite its strengths, our study has several limitations inherent to the NIS database. First, the use of administrative coding may introduce errors and misclassification, including missed cases that underwent POEM before 2016. Although we restricted our analysis to patients with a primary diagnosis of achalasia, the lack of clinical granularity in the NIS limits our ability to determine whether hospitalizations were specifically related to therapeutic intervention versus other factors such as symptom burden, comorbidities, or complications. Another key limitation is the absence of follow‐up data, which restricts our ability to assess the recurrence of symptoms, adverse events or readmission rates. We were also unable to evaluate the specific subtype of achalasia, which could have influenced management decisions. Furthermore, our analysis did not account for hospital preferences or regional variability in the adoption of POEM, which may have affected the observed trends. Lastly, we are unable to assess the trends in the outpatient management of achalasia which likely led to an underestimation of the use of PD and POEM which are most commonly outpatient procedures.

In conclusion, between 2011 and 2022, the proportion of hospitalized patients undergoing procedural management for achalasia has declined, reflecting a progressive shift toward minimally invasive approaches and a corresponding decrease in the use of conventional, higher‐morbidity surgeries. In this period, while the duration of inpatient stay has remained relatively stable, there have been increasing costs, mortality, and complication rates, which are likely due to an increase in the comorbidity profile of the patients admitted with achalasia. There is a need for broader adoption of minimally invasive procedures like POEM for managing achalasia, preferably early in the disease, while making a concerted effort to reduce complications and improve patient outcomes.

## Author Contributions

R.M.G.: Conceptualization, Methodology, Writing – Original Draft; A.S.: Formal Analysis, Resources; R.K.: Writing – Original Draft; S.R.: Writing – Review and Editing; A.R.: Writing – Review and Editing, Supervision

## Conflicts of Interest

The authors declare no conflicts of interest.

## Supporting information


**Tables S1–S4:** nmo70202‐sup‐0001‐TableS1‐S4.pdf.

## Data Availability

The data that support the findings of this study are openly available in HCUP at https://hcup‐us.ahrq.gov/nisoverview.jsp.
