# Supplementary material for: A Decade of Change: National Trends in Management and Outcomes of Achalasia Hospitalizations in the United States
Source: Neurogastroenterol Motil. 2025 Nov 3;37(12):e70202. doi: 10.1111/nmo.70202 (PMC12623282; doi:10.1111/nmo.70202)
Supplement: Supplementary file 1 — Tables S1–S4: nmo70202‐sup‐0001‐TableS1‐S4.pdf. [file NMO-37-e70202-s001.pdf]

**Supplementary Table 1: ICD-9 and ICD-10-CM and PCS Codes for procedures and outcomes studied**		
**Category**	**ICD-9 Codes**	**ICD-10 Codes**
**Laparoscopic Heller's Myotomy (LHM)**	42.7	0D840ZZ, 0D843ZZ, 0D844ZZ
**Peroral Endoscopic Myotomy (POEM)**	None	0D847ZZ, 0D848ZZ
**Esophageal Dilation**	42.92	0D737ZZ, 0D738ZZ, 0D747ZZ, 0D748ZZ, 0D737DZ, 0D738DZ, 0D747DZ, 0D748DZ
**Esophagectomy**	42.40, 42.41, 42.42	0DB50ZZ, 0DB53ZZ, 0DT50ZZ, 0DT54ZZ
**Pneumonia**	482.x, 483.8, 484.x, 486	J13, J14, J15, J16, J17, J18
**Aspiration Pneumonitis**	507.0	J69.0
**Respiratory Failure following Surgery**	997.31, 997.39	J95.4, J95.851
**Sepsis**	038.0-038.9	A40, A41
**Post-procedural Shock**	998.0	T81.1
**Severe Sepsis**	995.92	R652
**Respiratory Failure**	518.81	J9601
**Shock**	785.50, 785.59	R578, R579
**Hypoglycemia**	251.x	E16

Supplementary table 2: Proportion of patients with achalasia undergoing LHM, PD, Esophagectomy and POEM

Year	LHM percentage	PD Percentage	Esophagectomy percentage	POEM
2011	48.22	6.54	1.49	
2012	41.73	6.19	1.42	
2013	43.08	9.65	1.59	
2014	45.88	8.23	1.35	
2015	43.66	7.17	1.74	
2016	40.49	3.24	0.61	5.78
2017	35.45	3.35	0.35	8.84
2018	37.53	3.61	0.64	7.32
2019	31.87	4.93	0.62	7.45
2020	30.51	4.93	0.21	9.55
2021	30.99	5.54	0.52	9.32
2022	27.59	5.19	0.52	10.27

Abbreviation: LHM: Laparoscopic Heller’s Myotomy; PD: Pneumatic Dilatation; POEM: Per Oral Endoscopic Myotomy

Supplementary Table 3: Demographic, comorbidity profile, and outcomes of patients admitted with achalasia undergoing POEMs									
	Total	2016	2017	2018	2019	2020	2021	2022	p-value
**Number of POEM among Achalasia Admissions (N)**	**623**	**66**	**100**	**79**	**83**	**87**	**89**	**99**	
**Weighted N**	3015	330	500	395	415	435	445	495	
**Age (mean)**	57.95 ± 17.59	55.24 ± 17.65	56.64 ± 18.72	59.30 ± 17.56	59.84 ± 17.74	56.47 ± 16.54	59.69 ± 16.37	58.05 ± 18.19	0.54
**Female (%)**	50.75	34	51	39	38	45	51	48	0.85
**Race (%)**									0.063
Whites	68.73	79	69.5	63.6	65.4	69.4	77	60	
Blacks	14.43	11.3	17.9	16.9	13.6	12.9	12.6	14.7	
Hispanics	10.3	8.1	8.4	11.7	8.6	16.5	3.4	14.7	
Asian or Pacific Islander	2.41	0	0	2.6	7.4	1.2	2.3	3.2	
Others	4.12	1.6	4.2	5.2	4.9	0	4.6	7.4	
**CCI score, %**									0.111
0	61.69	57.6	68	54.4	65.1	74.7	55.1	55.6	
1	8.29	10.6	10	6.3	6	9.2	10.1	6.1	
2	11.11	15.2	6	16.5	12	5.7	9	15.2	
≥3	18.91	16.7	16	22.8	16.9	10.3	25.8	23.2	
**Insurance Type (%)**									0.655
Medicare	43.8	40.9	43	45.6	45.8	36.8	48.3	45.5	
Medicaid	10.0	13.6	10	7.6	12	10.3	5.6	11.1	
Private Insurance	40.8	43.9	41	40.5	37.4	50.6	39.3	34.3	
Other (Other includes self-pay, no charge, and other insurance types.)	5.5	1.5	6.0	6.3	4.8	2.3	6.7	9.1	
**Hospitalization charges (in USD)**	61992.45	51305.7	68400.31	65250.78	61013.63	55658.76	59414.87	68807.07	0.528

**Length of stay (in days)**	3.14	4.12	3.56	3.45	2.75	2.30	2.89	3.10	0.511

Supplementary Table 4: Demographic, comorbidity profile, and outcomes of patients admitted with achalasia undergoing Heller's Myotomy								
	Total	2011-12	2013-14	2015-16	2017-18	2019-2020	2021-2022	p-value
**Number of Hellers Myotomy among Achalasia Admissions (N)**	4,895	993	940	961	806	633	562	
**Weighted N**	24,331.094	4,821.1	4,700	4,805	4,030	3,165	2,809	
**Age (mean)**	54.84 ± 16.69	53.03 ± 16.37	53.23 ± 17.07	54.59 ± 16.84	55.84 ± 16.43	57.16 ± 16.34	57.11 ±16.52	<0.001
**Female (%)**	49.6	49.2	49.7	52.3	48.6	48.8	47.5	0.494
**Race (%)**								0.010
Whites	69.3	71.2	71.5	68.4	66.2	66.0	72.2	
Blacks	14.6	15.5	14.5	14.7	15.3	14.5	11.8	
Hispanics	10.2	8.5	9.2	9.7	12.3	12.4	9.6	
Asian/PI	2.2	2.0	0.6	2.6	2.7	2.6	2.9	
Others	3.9	2.8	4.2	4.7	3.5	4.5	3.5	
**CCI score, %**								<0.001
0	68.9	71.8	70.1	68.7	69.4	63.5	66.9	
1	8.9	11.4	13.6	7.8	5.5	6.2	6.4	
2	10.1	9.7	8.3	10.6	11.0	10.4	11.0	
≥3	12.2	7.1	8.0	12.9	14.1	19.9	15.7	
**Insurance Type (%)**								<0.001
Medicare	37.3	31.4	35.1	35.8	39.9	44.1	42.6	
Medicaid	10.7	9.2	8.3	11.4	11.9	12.9	12.3	
Private Insurance	45.8	52.8	49.9	47.8	43.1	37.3	36.9	
Self-pay	2.3	2.5	2.8	2.2	1.7	1.9	2.7	
Other	3.8	4.1	3.9	2.9	3.2	3.8	5.5	
**Hospitalization charges (in USD)**	56921.159	44317.0	46066.13	55102.08	60925.90	67412.86	82135.7	<0.001
**Length of stay (in days)**	3.04	3.19	3.06	3.11	2.91	2.94	2.89	0.68
